# Development of Novel Biocomposite Scaffold of Chitosan-Gelatin/Nanohydroxyapatite for Potential Bone Tissue Engineering Applications

**DOI:** 10.1186/s11671-016-1669-1

**Published:** 2016-11-07

**Authors:** Yang Dan, Ouyang Liu, Yong Liu, Yuan-Yuan Zhang, Shuai Li, Xiao-bo Feng, Zeng-wu Shao, Cao Yang, Shu-Hua Yang, Ji-bo Hong

**Affiliations:** 1 Department of Orthopaedic Surgery, Union Hospital, Tongji Medical College, Huazhong University of Science and Technology, 1277 JieFang Avenue, Wuhan, 430022 China; 2 Department of Otolaryngology—Head and Neck Surgery, Renmin Hospital, Wuhan University, Wuhan, 430060 China

**Keywords:** Bone tissue engineering, Scaffolds, Preosteoblast cells, Mineralization

## Abstract

In this study, a three-dimensional chitosan-gelatin/nanohydroxyapatite (ChG/nHaP) scaffold was successfully fabricated and characterized in terms of swelling, degradation, cell proliferation, cell attachment, and mineralization characterizations. The ChG/nHaP scaffold was fabricated with a mean pore size of 100–180 μm. Our results showed that the physicochemical and biological properties of the scaffolds were affected by the presence of HaP. The swelling and degradation characteristics of the ChG scaffold were remarkably decreased by the addition of HaP. On the other hand, the presence of HaP remarkably improved the MC3T3-E1 cell attachment and cell growth in the scaffold membrane. The biocompatible nature of the ChG/nHaP scaffold leads to the development of finely scaled mineral deposits on the scaffold membrane. Thus, HaP played an important role in improving the biological performance of the scaffold. Therefore, the ChG/nHaP scaffold could be applied as a suitable material for bone tissue engineering applications.

## Background

According to estimation, nearly 2.5 million bone-grafting procedures are performed every year across the world. Until now, surgeons and experts mainly use bone grafts or substitute materials [1, 2]. This is the beginning of bone tissue engineering wherein constant research is going to discover a novel nanomaterial that could be an ideal bone substitute [3]. It has been well known that the bone consists of inorganic materials such as hydroxyapatite and organic material such as collagen, osteocalcin, and osteopontin [4]. The inorganic components provide the brittleness and strength whereas the organic material provides the much need for elasticity. Furthermore, the extracellular matrix (ECM) of bone acts as a scaffolding material that allows the regeneration of bone [5, 6]. Therefore, single-material-based scaffolds would be inefficient in maintaining the strength and rigidity which are normally required. Moreover, it is utmost important to find out materials which possess bone conductive properties and exactly simulate the ECM of bone [7–9]. An ideal tissue engineering scaffold is the one which can exactly mimic the extracellular matrix environment [10]. In the present study, therefore, we have selected a unique combination of chitosan, gelatin, and hydroxyapatite to make a scaffold.

Chitosan is a naturally occurring polymer comprised of glucosamine and *N*-acetylglucosamine [11]. Chitosan is highly biocompatible in nature and easily degraded by the enzymes present in the human body. Chitosan offers multiple advantages such as accelerate tissue engineering, hemostasis in wound healing, and antimicrobial properties [12]. Furthermore, chitosan is osteoconductive which makes it one of the ideal choices for tissue regeneration [13]. However, as with all polymers, chitosan holds low mechanical properties which require that chitosan be mixed with other bone materials.

Gelatin which is a partial derived product of collagen comprised of Arg-Gly-Asp (RGD) sequences which is normally found in the ECM [14]. Therefore, it allows for cell adhesion, attachment, and cell spreading more easily in a biocompatible manner. Moreover, gelatin has low antigenicity, and therefore, it possesses vital information signals for various biological processes. It has been reported earlier that mixture of chitosan and gelatin effectively promotes cell differentiation and cell proliferation [15].

Hydroxyapatite (HaP) is one of the main inorganic components of the human bone. HaP possesses excellent properties for tissue regeneration such as osteoconductivity, non-toxic nature, and non-inflammatory characteristics which effectively promote cell differentiation and cell growth [16, 17]. Besides HaP is a natural component of the bone, addition of HaP provides the much needed mechanical strength to the scaffold which is very important for the bone tissue engineering applications. One of the prerequisites which recently surfaced is that surface characteristics will greatly affect the cell proliferation and cytoskeletal arrangement and cell differentiation. Therefore, we have employed a nano-HaP (nHaP) to make the tissue engineering scaffolds [11, 18].

Based on all these facts, it can be expected that if chitosan, gelatin, and nanohydroxyapatite are mixed, then it will be very ideal for cell differentiation as well as for sufficient mechanical strength. Therefore, the main aim of the present study was to fabricate a chitosan-gelatin-nHaP composite scaffold for specific tissue engineering applications. The pores and morphology of the scaffold were evaluated by means of scanning electron microscopy (SEM). The in vitro characteristics such as degradation and swelling study were performed in phosphate-buffered saline (PBS). The biological property of the composite scaffold in enhancing the cell growth was evaluated by MTT assay and live/dead assay. The biocompatibility of the scaffold was determined by growing the cells on the surface of the scaffold and then viewed by microscopic imaging. Finally, mineralization capacity of MC3T3-E1 on the tissue engineering scaffold was assessed by alizarin red assay. Overall, we presented an attempt to evaluate the suitability of composite scaffolds for the various bone tissue engineering applications.

## Methods

### Materials

Chitosan (85 % degree of deacetylation), gelatin, nanohydroxyapatite (nHaP), alizarin red, sodium hydroxide, and sodium bromide were purchased from Sigma-Aldrich, China. All other chemicals were of reagent grade and used without further purification.

### Preparation of Chitosan-Gelatin/Nanohydroxyapatite Composite Scaffold

The composite scaffold was prepared by dissolving chitosan powder in 0.5 % acetic acid solution to make it a concentration of 2 %. The gelatin (0.75 %) was then added to the chitosan solution and stirred at room temperature for 15 h. Separately, slurry of nanohydroxyapatite was prepared (1 %) and added to the chitosan-gelatin solution and additionally stirred for 24 h. The resultant solution was ultrasonicated for 2 h to reduce the particle size and cross-linked with 0.25 % of glutaraldehyde. The resultant solution was transferred to a 6-well plate and subjected to freeze drying for 48 h. The dried scaffolds were neutralized with 2 % NaOH and 4 % NaBr for 2 h and washed with distilled water. The scaffolds were then freeze dried once again and stored at 8 °C until further use. A similar scaffold without nHaP was also prepared for comparison.

### In Vitro Swelling Characterizations

The swelling characteristics of the ChG and ChG/HaP scaffolds were performed in phosphate-buffered saline (PBS, pH 7.4) at 37 °C. A known mass of dried scaffold was placed in PBS buffer, and at a predetermined time, the scaffold was removed and surfaces were cleared of water and weight was measured. The ratio of swelling was calculated using the following formula:

$$ \mathrm{Swelling}\ \mathrm{ratio} = \left(\mathrm{W}\mathrm{w}-\mathrm{W}\mathrm{o}\right)/\mathrm{W}\mathrm{o} $$

### In Vitro Degradation Study

The degradation characteristics of the ChG and ChG/nHaP scaffolds were performed in phosphate-buffered saline (PBS, pH 7.4) at 37 °C. For this purpose, the scaffolds were immersed in PBS buffer containing lysozyme (10,000 U/ml) and incubated for a specified period. Initial weight of scaffold was noted, and at a specific time point, the scaffold was removed, washed, and freeze dried and weight was noted. The degradation of the scaffold was noted from the following formula:

$$ \mathrm{Degradation}\ \% = \left(\mathrm{W}\mathrm{o}-\mathrm{W}\mathrm{t}\right)/\mathrm{W}\mathrm{o} \times 100 $$

$$ \mathrm{W}\mathrm{o}-\mathrm{Initial}\ \mathrm{weight}\ \mathrm{o}\mathrm{f}\ \mathrm{the}\ \mathrm{scaffold} $$

$$ \mathrm{W}\mathrm{t}-\mathrm{Weight}\ \mathrm{of}\ \mathrm{t}\mathrm{he}\ \mathrm{scaffold}\ \mathrm{at}\ \mathrm{respective}\ \mathrm{t}\mathrm{ime}\ \mathrm{point} $$

### In Vitro Cell Viability Assay

The MC3T3-E1 cells were cultured in alpha-Minimum Essential Medium (MEM) with 1-mM sodium pyruvate and 2-mM l-glutamine containing 10 % FBS and 1 % antibiotic mixture. The in vitro cell viability was determined by MTT assay. The cells (3 × 10^5^) were seeded on the scaffold which is immersed in the media and allowed to incubate for a predetermined time point. At a specific time period, the scaffold was treated with MTT solution (5 % solution in RPMI) and incubated for 4 h. During this time, MTT was oxidized by mitochondrial enzymes and the purple-colored formazan crystal was extracted by adding DMSO. The absorbance was calculated at 570 nm using a standard plate reader.

### Live Dead Assay

The cells were trypsinized and seeded on composite scaffolds. The cells were allowed to grow for 14 days. The cells were then washed two times with PBS and then treated with LIVE/DEAD® viability/cytotoxicity kit (Life Technologies of Brazil Ltda., São Paulo) according to the manufacturer’s specifications. The images were observed using an inverted microscope (NIKON).

### Cell Adhesion and Spreading Analysis

The proliferation and growth of cells in the scaffold was evaluated by SEM imaging. The cells were allowed to grow on the scaffold for 30 days under the presence of growth medium. The media were changed every 2 days once. At the end of the incubation period, the scaffolds containing cells were washed carefully with PBS and fixed with 2 % glutaraldehyde at 37 °C. The dried samples were then immersed in a series of gradient ethanol (10, 20, 40, 60, 80, and 100 %) and then vacuum-dried in a desiccator. The samples were made conductive by sputtering with an ultra-thin carbon coating at a very low deposition rate and analyzed by SEM (Hitachi S4200).

### Alizarin Red Staining for Mineral Deposition

The cells were allowed to grow on the scaffold for 30 days under the presence of the growth medium. The media were changed every 2 days once. At the end of the incubation period, the scaffolds containing cells were washed carefully with PBS. The mineralization of MC3T3-E1 was observed by carrying out alizarin red staining. The scaffold was washed with PBS and carefully fixed with ice-cold 70 % ethanol solution for 20 min at 30 °C. The scaffold was then stained with 0.1 % of alizarin red solution and incubated for 30 min at mild shaking. The samples were observed using a microscope.

### Statistical Analysis

The statistical analysis was performed using GraphPad Prism (Ver.5.0), and all the values were presented as mean ± standard deviation. *p* < 0.05 was considered statistically significant.

## Results and Discussion

### Fabrication of Tissue Engineering Scaffold

At present, no synthetic bone substitute is available that can match the strength of the natural human bone. In this regard, bone tissue engineering attracted the attention of several researchers to overcome the limitations of the present scenario. A potential strategy would be to simulate the conditions that can mimic the ECM and accelerate the osteogenesis. Moreover, it is utmost important to find out materials which possess bone conductive properties and exactly simulate the ECM of the bone. An ideal tissue engineering scaffold is the one which can exactly mimic the extracellular matrix environment. In the present study, therefore, we have selected a unique combination of chitosan, gelatin, and hydroxyapatite to make a scaffold. Each of the material selected has its own importance and could play a pivotal role in the bone tissue engineering.

**Fig. 1:**
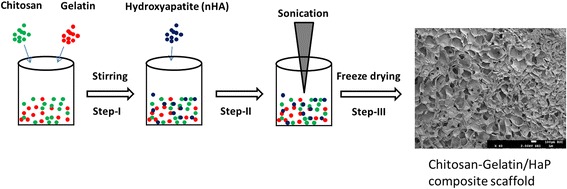
Schematic illustration of fabrication of chitosan-gelatin/hydroxyapatite (ChG/nHaP) scaffolds

The SEM examination revealed that composite scaffolds possessed extensive nanoscale pores which might provide additional or extra surface area for the growth of cells (Fig. 1). It should be noted that the pores are spread throughout the scaffold which may guarantee its performance. The surface of the scaffold was slightly rough due to the mixing of nHaP in the polymer mixture. The mean size of pores ranged from 80 to 180 μM. The presence of large pores in the scaffold helps the growth of cells while smaller pores will allow the easy passage of essential nutrients to the cells. In addition, smaller pores provide larger surface area and could play an important role in cell growth and proliferation.

### In Vitro Swelling Studies

**Fig. 2:**
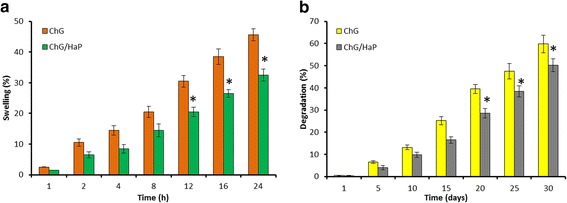
**a** In vitro swelling and **b** in vitro degradation studies of the ChG scaffold and ChG/nHaP scaffolds

In vitro swelling studies indicate a high swelling capacity for the chitosan-gelatin (ChG) and ChG/nanohydroxyapatite (nHaP) scaffolds (Fig. 2a). A high swelling capacity of the scaffold is proof that the scaffold material could absorb large volume of water than its original weight. It should be noted that the addition of HaP significantly decreased the swelling capacity of the scaffold. The decrease in swelling was attributed to the binding of nHaP to the chitosan and gelatin network. Specifically, calcium and phosphate of nHaP bind the COOH or NH2 group of chitosan/gelatin. The swelling of the scaffolds will facilitate the infiltration of cells in the scaffolds in a 3-D manner. Besides, swelling is also reported to increase the pore size wherein the internal surface area of the scaffold is expected to increase which will in turn allow cell infusion. The swelling would increase the cell attachment, and the cell growth would lower the mechanical strength. Therefore, although the ChG/nHaP scaffolds showed a lower swelling rate yet, it is expected to hold higher mechanical strength.

### In Vitro Degradation Studies

The degradation study was performed in the presence of a lysozyme—an enzyme primarily responsible for the degradation of chitosan backbone via hydrolysis of its residues (Fig. 2b). It can be seen that at the end of 30 days, the ChG scaffold exhibited nearly 60 % of degradation whereas approximately 50 % degradation was observed in the ChG/nHaP scaffolds. The decrease in the degradation capacity was due to the ability of HaP to reduce the access of enzymes to the polysaccharide moieties. The *N*-acetylglucosamine group of chitosan is prone to be degraded by the enzymes, and similarly, macromolecular chains of gelatin were also degrading in the presence of water. The addition of HaP however limits the degradation potential of polymer structure due to the limited access of enzyme. Nevertheless, a scaffold with moderate swelling and degradation characteristics with satisfactory mechanical strength would be ideal for tissue engineering applications.

### Cell Proliferation Analysis

**Fig. 3:**
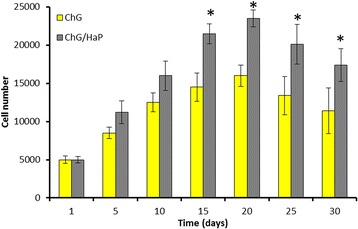
In vitro cell viability assay of MC3T3-E1 cells grown on the ChG scaffold and ChG/nHaP scaffolds

The proliferation of MC3T3-E1 cells on the composite scaffold was evaluated by MTT assay protocol (Fig. 3). As seen, both the scaffolds promoted the differentiation and proliferation of preosteoblast cells in an effective manner. To be specific, the ChG/nHaP scaffolds exhibited significantly higher cell numbers compared to the ChG scaffolds. The cell proliferation proportionately increased until day 15; however, after that, the cells were growing slowly, and in fact, they decreased after day 20. The reason for the decreased cell numbers after day 20 might be due to the coverage of all the free surface area available on the scaffold and lack of any new surface to grow. The ability of the ChG/nHaP scaffolds to allow higher growth might be due to the presence of minerals in the polymer backbone. Overall, results showed that the scaffolds were biocompatible and non-toxic in nature and would be suitable for the tissue engineering applications.

### Live/Dead Assay

**Fig. 4:**
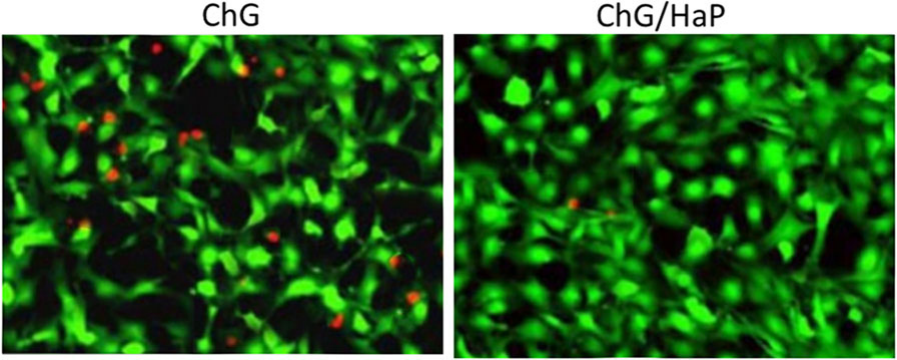
Live/dead assay of MC3T3-E1 cells grown on the ChG scaffold and ChG/nHaP scaffolds

Consistently, live/dead assay was performed to evaluate the proportion of live cells and dead cells (Fig. 4). As seen, both polymeric scaffolds did not hamper the proliferation of cells in any manner. The ChG/nHaP scaffolds showed bright green fluorescence compared to the ChG scaffolds with no presence of dead cells.

### Cell Morphology on the Scaffolds

**Fig. 5:**
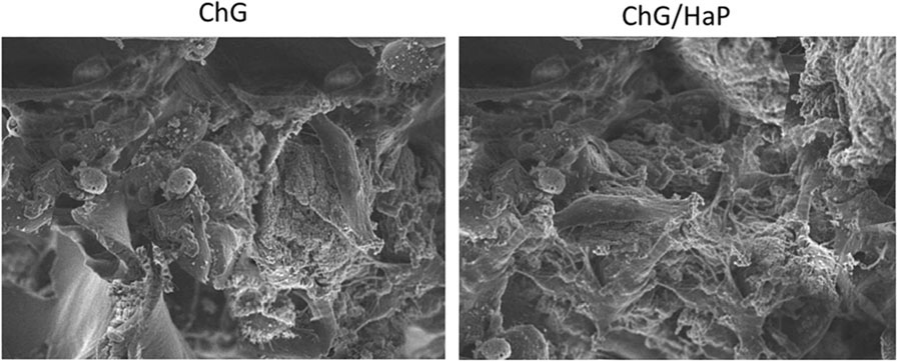
Cell adhesion study of MC3T3-E1 cells on the ChG scaffold and ChG/nHaP scaffolds

The cell morphology in the scaffold was assessed by SEM. The cell adhesion studies revealed that cells were well attached to the scaffold membrane and penetrated the pores of the scaffold after 30 days of incubation (Fig. 5). In case of the ChG scaffolds, we observed fewer cells attached on the surface of the scaffold while inner pores were deficient of any cell proliferations. In case of the ChG/nHaP scaffolds, we have observed a substantial amount of cell attachment and cell spreading. Specifically, it can be seen that cells penetrated the inner pores of the scaffold and present like a flattened sheet. The high attachment and spreading of the cells in the ChG/nHaP scaffolds was clearly attributed to the presence of HaP which facilitated the formation of focal adhesion and thereby cell proliferation. Therefore, it could be clearly concluded that the ChG/nHaP scaffolds would be more suitable for tissue engineering applications than the ChG scaffolds.

### In Vitro Mineralization Study

**Fig. 6:**
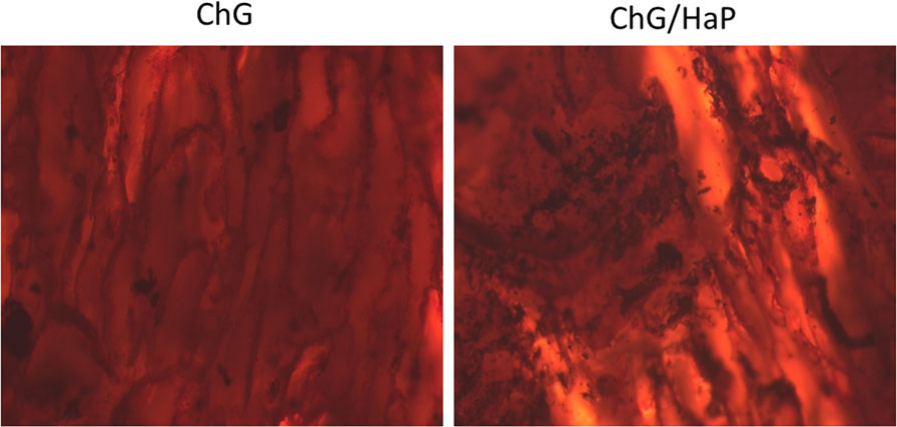
In vitro mineralization study of the ChG scaffold and ChG/nHaP scaffolds. The mineralization study was performed by alizarin red assay

The mineralization study was performed by alizarin red staining. After 30 days of cell proliferation, minerals were expected to be present on the scaffold membrane (Fig. 6). As seen, less mineralization of the scaffold was observed in the ChG scaffolds whereas remarkably higher mineralization was noted in the ChG/nHaP scaffolds, indicating the superior cytocompatibility of the HaP-containing scaffolds. This result suggests that the ChG/nHaP scaffolds offer excellent biocompatibility for bone regeneration. The biocompatible nature of the ChG/nHaP scaffold leads to the proliferation of cells that will lead to the development of finely scaled mineral deposits on the scaffold membrane. Therefore, the presence of essential minerals such as calcium is essential for the cell proliferation as well as for tissue regeneration. Such a mineralized scaffold can improve specific biological functions such as adhesion, differentiation, and proliferation of osteoblastic cells.

## Conclusions

In conclusion, a three-dimensional ChG/nHaP scaffold was successfully fabricated and characterized in terms of swelling, degradation, cell proliferation, cell attachment, and mineralization characterizations. The ChG/nHaP scaffold was fabricated with a mean pore size of 100–180 μm. Our results showed that the physicochemical and biological properties of the scaffolds were affected by the presence of HaP. The swelling and degradation characteristics of the ChG scaffold were remarkably decreased by the addition of HaP. On the other hand, the presence of HaP remarkably improved the MC3T3-E1 cell attachment and cell growth in the scaffold membrane. The biocompatible nature of the ChG/nHaP scaffold leads to the development of finely scaled mineral deposits on the scaffold membrane. Thus, HaP played an important role in improving the biological performance of the scaffold. Therefore, the ChG/nHaP scaffold could be applied as a suitable material for the bone tissue engineering applications.
